# ChatGPT Utility in Healthcare Education, Research, and Practice: Systematic Review on the Promising Perspectives and Valid Concerns

**DOI:** 10.3390/healthcare11060887

**Published:** 2023-03-19

**Authors:** Malik Sallam

**Affiliations:** 1 Department of Pathology, Microbiology and Forensic Medicine, School of Medicine, The University of Jordan, Amman 11942, Jordan; malik.sallam@ju.edu.jo; Tel.: +962-79-184-5186; 2 Department of Clinical Laboratories and Forensic Medicine, Jordan University Hospital, Amman 11942, Jordan

**Keywords:** machine learning, digital health, artificial intelligence, healthcare, ethics

## Abstract

ChatGPT is an artificial intelligence (AI)-based conversational large language model (LLM). The potential applications of LLMs in health care education, research, and practice could be promising if the associated valid concerns are proactively examined and addressed. The current systematic review aimed to investigate the utility of ChatGPT in health care education, research, and practice and to highlight its potential limitations. Using the PRIMSA guidelines, a systematic search was conducted to retrieve English records in PubMed/MEDLINE and Google Scholar (published research or preprints) that examined ChatGPT in the context of health care education, research, or practice. A total of 60 records were eligible for inclusion. Benefits of ChatGPT were cited in 51/60 (85.0%) records and included: (1) improved scientific writing and enhancing research equity and versatility; (2) utility in health care research (efficient analysis of datasets, code generation, literature reviews, saving time to focus on experimental design, and drug discovery and development); (3) benefits in health care practice (streamlining the workflow, cost saving, documentation, personalized medicine, and improved health literacy); and (4) benefits in health care education including improved personalized learning and the focus on critical thinking and problem-based learning. Concerns regarding ChatGPT use were stated in 58/60 (96.7%) records including ethical, copyright, transparency, and legal issues, the risk of bias, plagiarism, lack of originality, inaccurate content with risk of hallucination, limited knowledge, incorrect citations, cybersecurity issues, and risk of infodemics. The promising applications of ChatGPT can induce paradigm shifts in health care education, research, and practice. However, the embrace of this AI chatbot should be conducted with extreme caution considering its potential limitations. As it currently stands, ChatGPT does not qualify to be listed as an author in scientific articles unless the ICMJE/COPE guidelines are revised or amended. An initiative involving all stakeholders in health care education, research, and practice is urgently needed. This will help to set a code of ethics to guide the responsible use of ChatGPT among other LLMs in health care and academia.

## 1. Introduction

Artificial intelligence (AI) can be defined as the multidisciplinary approach of computer science and linguistics that aspires to create machines capable of performing tasks that normally require human intelligence [1]. These tasks include the ability to learn, adapt, rationalize, understand, and to fathom abstract concepts as well as the reactivity to complex human attributes such as attention, emotion, creativity, etc. [2].

The history of AI as a scientific discipline can be traced back to the mid-XX century at the Dartmouth Summer Research Project on AI [3]. This was followed by the development of machine learning (ML) algorithms that allow decision-making or predictions based on the patterns in large datasets [4]. Subsequently, the development of neural networks (brain-mimicking algorithms), genetic algorithms (finding optimal solutions for complex problems by application of evolutionary principles), and other advanced techniques followed [5].

Launched in November 2022, “ChatGPT” is an AI-based large language model (LLM) trained on massive text datasets in multiple languages with the ability to generate human-like responses to text input [6]. Developed by OpenAI (OpenAI, L.L.C., San Francisco, CA, USA), ChatGPT etymology is related to being a chatbot (a program able to understand and generate responses using a text-based interface) and is based on the generative pre-trained transformer (GPT) architecture [6,7]. The GPT architecture utilizes a neural network to process natural language, thus generating responses based on the context of input text [7]. The superiority of ChatGPT compared to its GPT-based predecessors can be linked to its ability to respond to multiple languages generating refined and highly sophisticated responses based on advanced modeling [6,7].

In the scientific community and academia, ChatGPT has received mixed responses reflecting the history of controversy regarding the benefits vs. risks of advanced AI technologies [8,9,10]. On one hand, ChatGPT, among other LLMs, can be beneficial in conversational and writing tasks, assisting to increase the efficiency and accuracy of the required output [11]. On the other hand, concerns have been raised in relation to possible bias based on the datasets used in ChatGPT training, which can limit its capabilities and could result in factual inaccuracies, but alarmingly appear to be scientifically plausible (a phenomenon termed hallucination) [11]. Additionally, security concerns and the potential of cyber-attacks with the spread of misinformation utilizing LLMs should also be considered [11].

The innate resistance of the human mind to any change is a well-described phenomenon and can be understandable from evolutionary and social psychology perspectives [12]. Therefore, the concerns and debate that arose instantaneously following the widespread release of ChatGPT appear to be understandable. The attention that ChatGPT received involved several disciplines. In education, for example, ChatGPT release could mark the end of essays as assignments [13]. In health care practice and academic writing, factual inaccuracies, ethical issues, and the fear of misuse including the spread of misinformation should be considered [14,15,16].

The versatility of human intelligence (HI) compared to AI is related to its biological evolutionary history, adaptability, creativity, the ability of emotional intelligence, and the ability to understand complex abstract concepts [2]. However, HI-AI cooperation can be beneficial if an accurate and reliable output of AI is ensured. The promising utility of AI in health care has been outlined previously with possible benefits in personalized medicine, drug discovery, and the analysis of large datasets aside from the potential applications to improve diagnosis and clinical decisions [17,18]. Additionally, the utility of AI chatbots in health care education is an interesting area to probe. This is related to the massive information and various concepts that health care students are required to grasp [19]. However, all of these applications should be considered cautiously considering the valid concerns, risks, and categorical failures experienced and cited in the context of LLM applications. Specifically, Borji comprehensively highlighted the caveats of ChatGPT use that included, but were not limited to, the generation of inaccurate content, the risk of bias and discrimination, lack of transparency and reliability, cybersecurity concerns, ethical consequences, and societal implications [20].

Therefore, the aim of the current review was to explore the future perspectives of ChatGPT as a prime example of LLMs in health care education, academic/scientific writing, health care research, and health care practice based on the existing evidence. Importantly, the current review objectives extended to involve the identification of potential limitations and concerns that could be associated with the application of ChatGPT in the aforementioned areas in health care settings.

## 2. Materials and Methods

### 2.1. Search Strategy and Inclusion Criteria

The current systematic review was conducted according to the Preferred Reporting Items for Systematic Reviews and Meta-Analyses (PRIMSA) guidelines [21]. The information sources included PubMed/MEDLINE and Google Scholar.

The eligibility criteria involved any type of published scientific research or preprints (article, review, communication, editorial, opinion, etc.) addressing ChatGPT that fell under the following categories: (1) health care practice/research; (2) health care education; and (3) academic writing.

The exclusion criteria included: (1) non-English records; (2) records addressing ChatGPT in subjects other than those mentioned in the eligibility criteria; and (3) articles from non-academic sources (e.g., newspapers, internet websites, magazines, etc.).

The exact PubMed/MEDLINE search strategy, which concluded on 16 February 2023, was as follows: (ChatGPT) AND ((“2022/11/30” [Date–Publication]: “3000” [Date–Publication])), which yielded 42 records.

The search on Google Scholar was conducted using Publish or Perish (Version 8) [22]. The search term was “ChatGPT” for the years 2022–2023, and the Google Scholar search yielded 238 records and concluded on 16 February 2023.

### 2.2. Summary of the Record Screening Approach

The records retrieved following the PubMed/MEDLINE and Google Scholar searches were imported to EndNote v.20 for Windows (Thomson ResearchSoft, Stanford, CA, USA), which yielded a total of 280 records.

Next, screening of the title/abstract was conducted for each record with the exclusion of duplicate records (*n* = 40), followed by the exclusion of records published in languages other than English (*n* = 32). Additionally, the records that fell outside the scope of the review (records that examined ChatGPT in a context outside health care education, health care practice, or scientific research/academic writing) were excluded (*n* = 80). Moreover, the records published in non-academic sources (e.g., newspapers, magazines, Internet websites, blogs, etc.) were excluded (*n* = 18).

Afterward, full screening of the remaining records (*n* = 110) was carried out with the exclusion of an additional 41 records that fell outside the scope of the current review. An additional nine records were excluded due to the inability to access the full text of these records, being subscription-based. This yielded a total of 60 records eligible for inclusion in the current review.

### 2.3. Summary of the Descriptive Search for ChatGPT Benefits and Risks in the Included Records

Each of the included records was searched specifically for the following: (1) type of record (preprint, published research article, opinion, commentary, editorial, review, etc.); (2) the listed benefits/applications of ChatGPT in health care education, health care practice, or scientific research/academic writing; (3) the listed risks/concerns of ChatGPT in health care education, health care practice, or scientific research/academic writing; and (4) the main conclusions and recommendations regarding ChatGPT in health care education, health care practice, or scientific research/academic writing.

Categorization of the benefits/applications of ChatGPT was as follows: (1) educational benefits in health care education (e.g., generation of realistic and variable clinical vignettes, customized clinical cases with immediate feedback based on the student’s needs, enhanced communications skills); (2) benefits in academic/scientific writing (e.g., text generation, summarization, translation, and literature review in scientific research); (3) benefits in scientific research (e.g., efficient analysis of large datasets, drug discovery, identification of potential drug targets, generation of codes in scientific research); (4) benefits in health care practice (e.g., improvements in personalized medicine, diagnosis, treatment, lifestyle recommendations based on personalized traits, documentation/generation of reports); and (5) being a freely available package.

Categorization of the risks/concerns of ChatGPT was as follows: (1) ethical issues (e.g., risk of bias, discrimination based on the quality of training data, plagiarism); (2) hallucination (the generation of scientifically incorrect content that sounds plausible); (3) transparency issues (black box application); (4) risk of declining need for human expertise with subsequent psychologic, economic and social issues; (5) over-detailed, redundant, excessive content; (6) concerns about data privacy for medical information; (7) risk of declining clinical skills, critical thinking and problem-solving abilities; (8) legal issues (e.g., copyright issues, authorship status); (9) interpretability issues; (10) referencing issues; (11) risk of academic fraud in research; (12) incorrect content; and (13) infodemic risk.

## 3. Results

**Figure 1:**
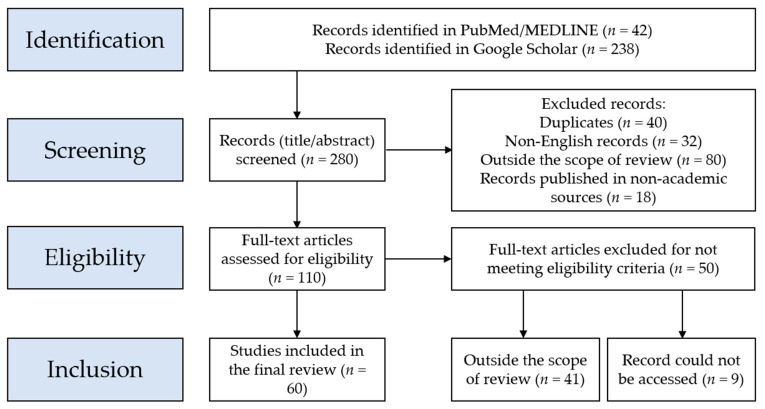
Flowchart of the record selection process based on the Preferred Reporting Items for Systematic Reviews and Meta-Analyses (PRIMSA) guidelines.

A total of 280 records were identified, and following the full screening process, a total of 60 records were eligible to be included in the review. The PRISMA flowchart of the record selection process is shown in Figure 1.

### 3.1. Summary of the ChatGPT Benefits and Limitations/Concerns in Health Care

Summaries of the main conclusions of the included studies regarding ChatGPT utility in academic writing, health care education, and health care practice/research are provided in Table 1 for the records comprising editorials/letters to the editors, in Table 2 for the records comprising research articles, commentaries, news articles, perspectives, case studies, brief reports, communications, opinions, or recommendations, and in Table 3 for the records representing preprints.

### 3.2. Characteristics of the Included Records

**Figure 2:**
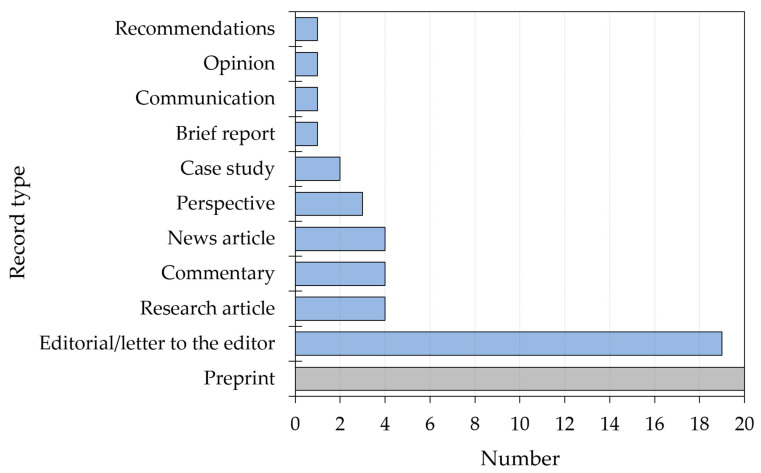
Summary of the types of included records (*n* = 60). Preprints (not peer reviewed) are highlighted in grey while published records are highlighted in blue.

A summary of the record types included in the current review is shown in Figure 2.

One-third of the included records were preprints (*n* = 20), with the most common preprint server being medRxiv (*n* = 6, 30.0%), followed by SSRN and arXiv (*n* = 4, 20.0%) for each. Editorials/letters to editors were the second most common type of included records (*n* = 19, 31.7%).

### 3.3. Benefits and Possible Applications of ChatGPT in Health Care Education, Practice, and Research Based on the Included Records

**Figure 3:**
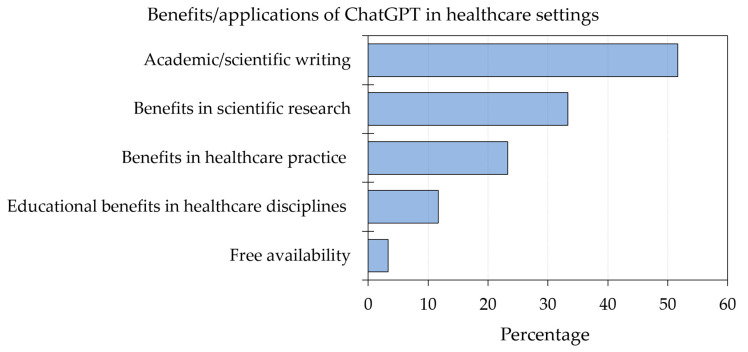
Summary of benefits/applications of ChatGPT in health care education, research, and practice based on the included records.

The benefits of ChatGPT were most frequently cited in the context of academic/scientific writing, which was mentioned in 31 records (51.7%). Examples included efficiency and versatility in writing with text of high quality, improved language, readability, and translation promoting research equity, and accelerated literature review. Benefits in scientific research followed, which was mentioned in 20 records (33.3%). Examples included the ability to analyze massive data including electronic health records or genomic data, the availability of more free time for the focus on experimental design, and drug design and discovery. Benefits in health care practice was mentioned by 14 records (23.3%), with examples including personalized medicine, prediction of disease risk and outcome, streamlining the clinical workflow, improved diagnostics, documentation, cost saving, and improved health literacy. Educational benefits in health care disciplines were mentioned in seven records (11.7%) with examples including the generation of accurate and versatile clinical vignettes, improved personalized learning experience, and being an adjunct in group learning. Being a free package was mentioned as a benefit in two records (3.3%, Figure 3).

### 3.4. Risks and Concerns toward ChatGPT in Health Care Education, Practice, and Research Based on the Included Records

Ethical concerns were commonly mentioned by 33 records (55.0%), especially in the context of risk of bias (mentioned by 18 records, 30.0%) and plagiarism (mentioned by 14 records, 23.3%) among data privacy and security issues.

**Figure 4:**
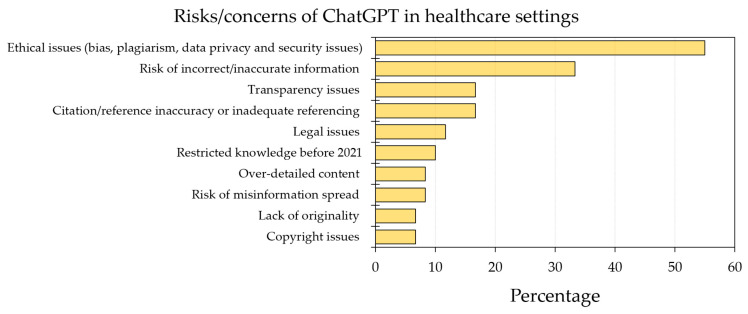
Summary of risks/concerns of ChatGPT use in health care education, research, and practice based on the included records.

Other concerns involved the risk of incorrect/inaccurate information, which was mentioned by 20 records (33.3%); citation/reference inaccuracy or inadequate referencing, which was mentioned by 10 records (16.7%); transparency issues, which was mentioned by 10 records (16.7%); legal issues were mentioned in seven records (11.7%); restricted knowledge before 2021 was mentioned by six records (10.0%); risk of misinformation spread was mentioned by five records (8.3%); over-detailed content was mentioned in five records (8.3%); copyright issues were mentioned in four records (6.7%); and the lack of originality was mentioned by four records (6.7%, Figure 4).

## 4. Discussion

The far-reaching consequences of ChatGPT among other LLMs can be described as a paradigm shift in academia and health care practice [16]. The discussion of its potential benefits, future perspectives, and importantly, its limitations, appear timely and relevant [80].

Therefore, the current review aimed to highlight these issues based on the current evidence. The following common themes emerged from the available literature.

### 4.1. Benefits of ChatGPT in Scientific Research

ChatGPT, as an example of other LLMs, can be described as a promising or even a revolutionary tool for scientific research in both academic writing and in the research process itself. Specifically, ChatGPT was listed in several sources as an efficient and promising tool for conducting comprehensive literature reviews and generating computer codes, thereby saving time for the research steps that require more efforts from human intelligence (e.g., the focus on experimental design) [14,27,28,41,44,46,47,60,71,72]. Additionally, ChatGPT can be helpful in generating queries for comprehensive systematic review with high precision, as shown by Wang et al., despite the authors highlighting the transparency issues and unsuitability for high-recall retrieval [61]. Moreover, the utility of ChatGPT extends to involve an improvement in language and a better ability to express and communicate research ideas and results, ultimately speeding up the publication process with the faster availability of research results [23,25,29,40,48,63,66]. This is particularly relevant for researchers who are non-native English speakers [23,25,63]. Such a practice can be acceptable considering the already existent English editing services provided by several academic publishers. Subsequently, this can help to promote equity and diversity in research [46,55].

### 4.2. Limitations of ChatGPT Use in Scientific Research

On the other hand, the use of ChatGPT in academic writing and scientific research should be conducted in light of several limitations that could compromise the quality of research as follows. First, superficial, inaccurate, or incorrect content was frequently cited as a shortcoming of ChatGPT use in scientific writing [14,28,29,40,60]. The ethical issues including the risk of bias based on training datasets and plagiarism were also frequently mentioned, aside from the lack of transparency regarding content generation, which justifies the description of ChatGPT, on occasions, as a black box technology [14,25,26,40,44,45,46,47,48,55,60,63,65,72]. Importantly, the concept of ChatGPT hallucination could be risky if the generated content is not thoroughly evaluated by researchers and health providers with proper expertise [37,56,73,77,79]. This comes in light of the ability of ChatGPT to generate incorrect content that appears plausible from a scientific point of view [81].

Second, several records mentioned the current problems regarding citation inaccuracies, insufficient references, and ChatGPT referencing to non-existent sources [23,26]. This was clearly shown in two recently published case studies with ChatGPT use in a journal contest [29,49,50]. These case studies discouraged the use of ChatGPT, citing the lack of scientific accuracy, the limited updated knowledge, and the lack of ability to critically discuss the results [38,49,50]. Therefore, the ChatGPT generated content, albeit efficient, should be meticulously examined prior to its inclusion in any research manuscripts or proposals for research grants.

Third, the generation of non-original, over-detailed, or excessive content can be an additional burden for researchers who should carefully supervise the ChatGPT-generated content [14,24,25,26,65,71]. This can be addressed by supplying ChatGPT with proper prompts (text input), since varying responses might be generated based on the exact approach of prompt construction [72,82].

Fourth, as it currently stands, the knowledge of ChatGPT is limited to the period prior to 2021 based on the datasets used in ChatGPT training [6]. Thus, ChatGPT cannot be used currently as a reliable updated source of literature review [83]. Nevertheless, ChatGPT can be used as a motivation to organize the literature in a decent format, if supplemented by reliable and up-to-date references [28,74].

Fifth, the risk of research fraud (e.g., ghostwriting, falsified or fake research) involving ChatGPT should be considered seriously [23,37,38,66,72,79] as well as the risk of generating mis- or disinformation with the subsequent possibility of infodemics [26,46,48,66].

Sixth, legal issues in relation to ChatGPT use were also raised by several records including copyright issues [14,38,44,55,79]. Finally, the practice of listing ChatGPT as an author does not appear to be acceptable based on the current ICMJE and COPE guidelines for determining authorship, as illustrated by Zielinski et al. and Liebrenz et al. [43,48]. This comes in light of the fact that authorship entails legal obligations that are not met by ChatGPT [43,48]. However, other researchers have suggested the possibility of ChatGPT inclusion as an author in some specified instances [60,64].

A few instances were encountered in this review, where ChatGPT was listed as an author that can point to the initial perplexity of a few publishers regarding the role of LLM including ChatGPT in research [36,54]. The disapproval of including ChatGPT or any other LLM in the list of authors was clearly explained in *Science*, *Nature*, and the *Lancet* editorials, which referred to such practice as scientific misconduct, and this view was echoed by many scientists [24,27,35,40,45]. In the case of ChatGPT use in the research process, several records advocated the need for the proper and concise disclosure and documentation of ChatGPT or LLM use in the methodology or acknowledgement sections [35,63,65]. A noteworthy and comprehensive record by Borji can be used as a categorical guide for the issues and concerns of ChatGPT use, especially in the context of scientific writing [20].

### 4.3. Benefits of ChatGPT in Health Care Practice

From the health care practice perspective, the current review showed a careful excitement vibe regarding ChatGPT applications. The ability of ChatGPT to help in streamlining the clinical workflow appears promising, with possible cost savings and increased efficiency in health care delivery [31,37,39,77]. This was illustrated recently by Patel and Lam, highlighting the ability of ChatGPT to produce efficient discharge summaries, which can be valuable to reduce the burden of documentation in health care [53]. Additionally, ChatGPT, among other LLMs, can have a transforming potential in health care practice via enhancing diagnostics, prediction of disease risk and outcome, and drug discovery among other areas in translational research [51,52,68]. Moreover, ChatGPT showed moderate accuracy in determining the imaging steps needed in breast cancer screening and in the evaluation of breast pain, which can be a promising application in decision making in radiology [69]. ChatGPT in health care settings also has the prospects of refining personalized medicine and the ability to improve health literacy by providing easily accessible and understandable health information to the general public [30,32,59,73,74]. This utility was demonstrated by ChatGPT responses, highlighting the need to consult health care providers among other reliable sources on specific situations [16,54].

### 4.4. Concerns Regarding ChatGPT Use in Health Care Practice

On the other hand, several concerns regarding ChatGPT use in health care settings were raised. Ethical issues including the risk of bias and transparency issues appeared as recurring major concerns [51,68,69,77]. Additionally, the generation of inaccurate content can have severe negative consequences in health care; therefore, this valid concern should be cautiously considered in health care practice [30,32,53,84]. This concern also extends to involve the ability of ChatGPT to provide justification for incorrect decisions [69].

Other ChatGPT limitations including the issues of interpretability, reproducibility, and the handling of uncertainty were also raised, which can have harmful consequences in health care settings including health care research [68,72,73]. In the area of personalized medicine, the lack of transparency and unclear information regarding the sources of data used for ChatGPT training are important issues in health care settings considering the variability observed among different populations in several health-related traits [69]. The issue of reproducibility between the ChatGPT prompt runs is of particular importance, which can be a major limitation in health care practice [51].

Medico-legal and accountability issues in the case of medical errors caused by ChatGPT application should be carefully considered [44]. Importantly, the current LLMs including ChatGPT are unable to comprehend the complexity of biologic systems, which is an important concept needed in health care decisions and research [52,68]. The concerns regarding data governance, health care cybersecurity, and data privacy should draw specific attention in the discussion regarding the utility of LLMs in health care [32,39,53].

Other issues accompanying ChatGPT applications in health care include the lack of personal and emotional perspectives needed in health care delivery and research [30,55]. However, ChatGPT emulation of empathetic responses was reported in a preprint in the context of hepatic disease [74]. Additionally, the issue of devaluing the function of the human brain should not be overlooked; therefore, stressing the indispensable human role in health care practice and research is important to address any psychologic, economic, and social consequences that could accompany the application of LLM tools in health care settings [72].

### 4.5. Benefits and Concerns Regarding ChatGPT Use in Health Care Education

In the area of health care education, ChatGPT appears to have a massive transformative potential. The need to rethink and revise the current assessment tools in health care education comes in light of ChatGPT’s ability to pass reputable exams (e.g., USMLE) and possibility of ChatGPT misuse, which would result in academic dishonesty [24,34,58,59,62,76,85,86,87].

Specifically, in ophthalmology examination, Antaki et al. showed that ChatGPT currently performed at the level of an average first-year resident [70]. Such a result highlights the need to focus on questions involving the assessment of critical and problem-based thinking [34]. Additionally, the utility of ChatGPT in health care education can involve tailoring education based on the needs of the student with immediate feedback [46]. Interestingly, a recent preprint by Benoit showed the promising potential of ChatGPT in rapidly crafting consistent realistic clinical vignettes of variable complexities that can be a valuable educational source with lower costs [67]. Thus, ChatGPT can be useful in health care education including enhanced communication skills given proper academic mentoring [42,57,67]. However, the copyright issues should be taken into account regarding the ChatGPT-generated clinical vignettes, aside from the issue of inaccurate references [67]. Additionally, ChatGPT availability can be considered as a motivation in health care education based on the personalized interaction it provides, enabling powerful self-learning as well as its utility as an adjunct in group learning [30,33,36,57,58].

Other limitations of ChatGPT use in health care education include the concern regarding the quality of training datasets that could result in biased content and inaccurate information limited to the period prior to the year 2021. Additionally, other concerns include the current inability of ChatGPT to handle images as well as its low performance in some topics (e.g., failure to pass a parasitology exam for Korean medical students), and the issue of possible plagiarism [33,56,57,58,70,75]. Despite ChatGPT versatility in the context of academic education [79], the content of ChatGPT in research assignments was discouraged, being currently insufficient, biased, or misleading [36,78].

### 4.6. Future Perspectives

As stated comprehensively in a commentary by van Dis et al., there is an urgent need to develop guidelines for ChatGPT use in scientific research, taking into account the issues of accountability, integrity, transparency, and honesty [46,88]. Thus, the application of ChatGPT to advance academia and health care should be carried out ethically and responsibly, taking into account the potential risks and concerns it entails [47,89].

More studies are needed to evaluate the content of LLMs including its potential impact to advance academia and science with a particular focus on health care settings [90]. In academic writing, a question arises as to whether authors would prefer an AI-editor and an AI-reviewer considering the previous flaws in the editorial and peer review processes [91,92,93]. A similar question would also arise in health care settings involving the personal preference of emotional support from health care providers, rather than the potential efficiency of AI-based systems.

In health care education, more studies are needed to evaluate the potential impact of ChatGPT on the quality and efficiency of both educational content and assessment tools. ChatGPT utility to help in refining communication skills among health care students is another aspect that should be further explored as well as the applications of LLMs in the better achievement of the intended learning outcomes through personalized and instantaneous feedback for the students.

### 4.7. Strengths and Limitations

The current review represents the first rapid and concise overview of ChatGPT utility in health care education, research, and practice. However, the results of the current review should be viewed carefully in light of several shortcomings that include: (1) the quality of the included records can be variable, compromising the generalizability of the results; (2) the exclusion of non-English records might have resulted in selection bias; (3) the exclusion of several records that could not be accessed could have resulted in missing relevant data despite being small in number; (4) the inclusion of preprints that have not been peer reviewed but might also compromise the generalizability of the results; (5) the swift growth of literature addressing ChatGPT applications/risks mandate the need for further studies and reviews considering that the search in this review was concluded on 16 February 2023; and (6) this systematic review was based on the screening and interpretation of a single author, which may limit the interpretability of the results; therefore, future systematic reviews should consider collaborative work to improve the quality and credibility of the review results.

## 5. Conclusions

The imminent dominant use of LLM technology including the widespread use of ChatGPT in health care education, research, and practice is inevitable. Considering the valid concerns raised regarding its potential misuse, appropriate guidelines and regulations are urgently needed with the engagement of all stakeholders involved to ensure the safe and responsible use of ChatGPT powers. The proactive embrace of LLM technologies with careful consideration of the possible ethical and legal issues can limit the potential future complications. If properly implemented, ChatGPT, among other LLMs, have the potential to expedite innovation in health care and can aid in promoting equity and diversity in research by overcoming language barriers. Therefore, a science-driven debate regarding the pros and cons of ChatGPT is strongly recommended and its possible benefits should be weighed with the possible risks of misleading results and fraudulent research [94].

Based on the available evidence, health care professionals could be described as carefully enthusiastic regarding the huge potential of ChatGPT among other LLMs in clinical decision-making and optimizing the clinical workflow. “ChatGPT in the Loop: Humans in Charge” can be the proper motto to follow based on the intrinsic value of human knowledge and expertise in health care research and practice [14,25,55]. An inspiring example of this motto could be drawn based on the relationship between the human character Cooper and the robotic character TARS from Christopher Nolan’s movie Interstellar [95].

However, before its widespread adoption, the impact of ChatGPT from the health care perspective in a real-world setting should be conducted (e.g., using a risk-based approach) [96]. Based on the title of an important perspective article “AI in the hands of imperfect users” by Kostick-Quenet and Gerke [96], the real-world impact of ChatGPT among other LLMs should be properly evaluated to prevent any negative impact of its potential misuse. The same innovative and revolutionary tool can be severely deleterious if used improperly. An example to illustrate such severe negative consequences of ChatGPT misuse can be based on Formula 1 racing, as follows. In the 2004 Formula 1 season, the Ferrari F2004 (the highly successful Formula 1 racing car) broke several Formula 1 records in the hands of Michael Schumacher, one of the most successful Formula 1 drivers of all time. However, in my own hands —as a humble researcher without expertise in Formula 1 driving— the same highly successful car would only break walls and be damaged beyond repair.

## Figures and Tables

**Table 1:** A summary of the main conclusions of the included records comprising editorials/letters to the editors.

Author(s) [Record]	Design, Aims	Applications, Benefits	Risks, Concerns, Limitations	Suggested Action, Conclusions
Chen [23]	Editorial on ChatGPT applications in scientific writing	ChatGPT helps to overcome language barriers promoting equity in research	Ethical concerns (ghostwriting); doubtful accuracy; citation problems	Embrace this innovation with an open mind; authors should have proper knowledge on how to exploit AI ^6^ tools
Thorp [24]	Editorial: “ChatGPT is not an author”	-	Content is not original; incorrect answers that sound plausible; issues of referencing; risk of plagiarism	Revise assignments in education In *Science* journals, the use of ChatGPT is considered as a scientific misconduct
Kitamura [25]	Editorial on ChatGPT and the future of medical writing	Improved efficiency in medical writing; translation purposes	Ethical concerns, plagiarism; lack of originality; inaccurate content; risk of bias	“AI in the Loop: Humans in Charge”
Lubowitz [26]	Editorial, ChatGPT impact on medical literature	-	Inaccurate content; risk of bias; spread of misinformation and disinformation; lack of references; redundancy in text	Authors should not use ChatGPT to compose any part of scientific submission; however, it can be used under careful human supervision to ensure the integrity and originality of the scientific work
*Nature* [27]	*Nature* editorial on the rules of ChatGPT use to ensure transparent science	ChatGPT can help to summarize research papers; to generate helpful computer codes	Ethical issues; transparency issues	LLM ^7^ tools will be accepted as authors; if LLM tools are to be used, it should be documented in the methods or acknowledgements; advocate for transparency in methods, and integrity and truth from researchers
Moons and Van Bulck [28]	Editorial on ChatGPT potential in cardiovascular nursing practice and research	ChatGPT can help to summarize a large text; it can facilitate the work of researchers; it can help in data collection	Information accuracy issues; the limited knowledge up to the year 2021; limited capacity	ChatGPT can be a valuable tool in health care practice
Cahan and Treutlein [29]	Editorial reporting a conversation with ChatGPT on stem cell research	ChatGPT helps to save time	Repetition; several ChatGPT responses lacked depth and insight; lack of references	ChatGPT helped to write an editorial saving valuable time
Ahn [30]	A letter to the editor reporting a conversation of ChatGPT regarding CPR ^1^	Personalized interaction; quick response time; it can help to provide easily accessible and understandable information regarding CPR to the general public	Inaccurate information might be generated with possible serious medical consequences	Explore the potential utility of ChatGPT to provide information and education on CPR
Gunawan [31]	An editorial reporting a conversation with ChatGPT regarding the future of nursing	ChatGPT can help to increase efficiency; it helps to reduce errors in care delivery	Lack of emotional and personal support	ChatGPT can provide valuable perspectives in health care
D’Amico et al. [32]	An editorial reporting a conversation of ChatGPT regarding incorporating Chatbots into neurosurgical practice and research	ChatGPT can help to provide timely and accurate information for the patients about their treatment and care	Possibility of inaccurate information; privacy concerns; ethical issues; legal issues; risk of bias	Neurosurgery practice can be leading in utilizing ChatGPT into patient care and research
Fijačko et al. [33]	A letter to the editor to report the accuracy of ChatGPT responses with regards to life support exam questions by the AHA ^2^	ChatGPT provides relevant and accurate responses on occasions	Referencing issues; over-detailed responses	ChatGPT did not pass any of the exams; however, it can be a powerful self-learning tool to prepare for the life support exams
Mbakwe et al. [34]	An editorial on ChatGPT ability to pass the USMLE ^3^	-	Risk of bias; lack of thoughtful reasoning	ChatGPT passing the USMLE revealed the deficiencies in medical education and assessment; there is a need to reevaluate the current medical students’ training and educational tools
Huh [35]	An editorial of JEEHP ^4^ policy towards ChatGPT use	-	Reponses were not accurate in some areas	JEEHP will not accept ChatGPT as an author; however, ChatGPT content can be used if properly cited and documented
O’Connor * [36]	An editorial written with ChatGPT assistance on ChatGPT in nursing education	ChatGPT can help to provide a personalized learning experience	Risk of plagiarism; biased or misleading results	Advocate ethical and responsible use of ChatGPT; improve assessment in nursing education
Shen et al. [37]	An editorial on ChatGPT strengths and limitations	ChatGPT can help in the generation of medical reports; providing summaries of medical records; drafting letters to the insurance provider; improving the interpretability of CAD ^5^ systems	Risk of hallucination (inaccurate information that sounds plausible scientifically); the need to carefully construct ChatGPT prompts; possible inaccurate or incomplete results; dependence on the training data; risk of bias; risk of research fraud	Careful use of ChatGPT is needed to exploit its powerful applications
Gordijn and Have [38]	Editorial on the revolutionary nature of ChatGPT	-	Risk of factual inaccuracies; risk of plagiarism; risk of fraud; copyright infringements possibility	In the near future, LLM can write papers with the ability to pass peer review; therefore, the scientific community should be prepared to address this serious issue
Mijwil et al. [39]	An editorial on the role of cybersecurity in the protection of medical information	Versatility; efficiency; high quality of text generated; cost saving; innovation potential; improved decision making; improved diagnostics; predictive modeling; improved personalized medicine; streamline clinical workflow increasing efficiency; remote monitoring	Data security issues	The role of cybersecurity to protect medical information should be emphasized
*The Lancet Digital Health* [40]	An editorial on the strengths and limitations of ChatGPT	ChatGPT can help to improve language and readability	Over-detailed content; incorrect or biased content; potential to generate harmful errors; risk of spread of misinformation; risk of plagiarism; issues with integrity of scientific records	Widespread use of ChatGPT is inevitable; proper documentation of ChatGPT use is needed; ChatGPT should not be listed or cited as an author or co-author
Aljanabi et al. [41]	An editorial on the possibilities provided by ChatGPT	ChatGPT can help in academic writing; helpful in code generation	Inaccurate content including inability handle mathematical calculations to reliably	ChatGPT will receive a growing interest in the scientific community

^1^ CPR: cardiopulmonary resuscitation; ^2^ AHA: American Heart Association; ^3^ USMLE: United States Medical Licensing Examination; ^4^ JEEHP: Journal of Educational Evaluation for Health Professions; ^5^ CAD: computer-aided diagnosis; ^6^ AI: artificial intelligence; ^7^ LLM: large-scale language model. * ChatGPT generative pre-trained transformer was listed as an author.

**Table 2:** A summary of the main conclusions of the included records comprising research articles, commentaries, news articles, perspectives, case studies, brief reports, communications, opinions, or recommendations.

Author(s) [Record]	Design, Aims	Applications, Benefits	Risks, Concerns, Limitations	Suggested Action, Conclusions
Stokel-Walker [13]	News explainer	Well-organized content with decent references; a free package	Imminent end of conventional educational assessment; concerns regarding the effect on human knowledge and ability	The need to revise educational assessment tools to prioritize critical thinking or reasoning
Kumar [42]	Brief report; assessment of ChatGPT for academic writing in biomedicine	Original, precise, and accurate responses with systematic approach; helpful for training and to improving topic clarity; efficiency in time; promoting motivation to write	Failure to follow the instructions correctly on occasions; failure to cite references in-text; inaccurate references; lack of practical examples; lack of personal experience highlights; superficial responses	ChatGPT can help in improving academic writing skills; advocate for universal design for learning; proper use of ChatGPT under academic mentoring
Zielinski et al. [43]	WAME ^1^ recommendations on ChatGPT	ChatGPT can be a useful tool for researchers	Risk of incorrect or non-sensical answers; restricted knowledge to the period before 2021; lack of legal personality; risk of plagiarism	ChatGPT does not meet ICMJE ^4^ criteria and cannot be listed as an author; authors should be transparent regarding ChatGPT use and take responsibility for its content; editors need appropriate detection tools for ChatGPT-generated content
Biswas [44]	A perspective record on the future of medical writing in light of ChatGPT	Improved efficiency in medical writing	Suboptimal understanding of the medical field; ethical concerns; risk of bias; legal issues; transparency issues	A powerful tool in the medical field; however, several limitations of ChatGPT should be considered
Stokel-Walker [45]	A news article on the view of ChatGPT as an author	-	Risk of plagiarism; lack of accountability; concerns about misuse in the academia	ChatGPT should not be considered as an author
van Dis et al. [46]	A commentary on the priorities for ChatGPT research	ChatGPT can help to accelerate innovation; to increase efficiency in publication time; it can make science more equitable and increase the diversity of scientific perspectives; more free time for experimental designs; it could optimize academic training	Compromised research quality; transparency issues; risk of spread of misinformation; inaccuracies in content, risk of bias and plagiarism; ethical concerns; possible future monopoly; lack of transparency	ChatGPT ban will not work; develop rules for accountability, integrity, transparency and honesty; carefully consider which academic skills remain essential to researchers; widen the debate in the academia; an initiative is needed to address the development and responsible use of LLM ^5^ for research
Lund and Wang [47]	News article on ChatGPT impact in academia	Useful for literature review; can help in data analysis; can help in translation	Ethical concerns, issues about data privacy and security; risk of bias; transparency issues	ChatGPT has the potential to advance academia; consider how to use ChatGPT responsibly and ethically
Liebrenz et al. [48]	A commentary on the ethical issues of ChatGPT use in medical publishing	ChatGPT can help to overcome language barriers	Ethical issues (copyright, attribution, plagiarism, and authorship); inequalities in scholarly publishing; risk of spread of misinformation; inaccurate content	Robust AI authorship guidelines are needed in scholarly publishing; COPE AI ^6^ should be followed; AI cannot be listed as an author and it must be properly acknowledged upon its use
Manohar and Prasad [49]	A case study written with ChatGPT assistance	ChatGPT helped to generate a clear, comprehensible text	Lack of scientific accuracy and reliability; citation inaccuracies	ChatGPT use is discouraged due to risk of false information and non-existent citations; it can be misleading in health care practice
Akhter and Cooper [50]	A case study written with ChatGPT assistance	ChatGPT helped to provide a relevant general introductory summary	Inability to access relevant literature; the limited knowledge up to 2021; citation inaccuracies; limited ability to critically discuss results	Currently, ChatGPT cannot replace independent literature reviews in scientific research
Holzinger et al. [51]	An article on AI ^2^/ChatGPT use in biotechnology	Biomedical image analysis; diagnostics and disease prediction; personalized medicine; drug discovery and development	Ethical and legal issues; limited data availability to train the models; the issue of reproducibility of the runs	The scientists aspire for fairness, open science, and open data
Mann [52]	A perspective on ChatGPT in translational research	Efficiency in writing; analysis of large datasets (e.g., electronic health records or genomic data); predict risk factors for disease; predict disease outcomes	Compromised quality of data available; inability to understand the complexity of biologic systems	ChatGPT use in scientific and medical journals is inevitable in near future
Patel and Lam [53]	A commentary on ChatGPT utility in documentation of discharge summary	ChatGPT can help to reduce the burden of discharge summaries providing high-quality and efficient output	Data governance issues; risk of depersonalization of care; risk of incorrect or inadequate information	Proactive adoption of ChatGPT is needed to limit any possible future issues and limitations
Zhavoronkov * [54]	A perspective reporting a conversation with ChatGPT about rapamycin use from a philosophical perspective	ChatGPT provided correct summary of rapamycin side effects; it referred to the need to consult a health care provider based on the specific situation	-	Demonstration of ChatGPT’s potential to generate complex philosophical arguments
Hallsworth et al. [55]	A comprehensive opinion article submitted before ChatGPT launching on the value of theory-based research	It can help to circumvent language barriers; it can robustly help to process massive data in short time; it can stimulate creativity by humans if “AI in the Loop: Humans in Charge” is applied	Ethical issues; legal responsibility issues; lack of empathy and personal communication; lack of transparency	Despite the AI potential in science, there is an intrinsic value of human engagement in the scientific process which cannot be replaced by AI contribution
Stokel-Walker and Van Noorden [14]	*Nature* news feature article on ChatGPT implications in science	More productivity among researchers	Problems in reliability and factual inaccuracies; misleading information that seem plausible (hallucination); over-detailed content; risk of bias; ethical issues; copyright issues	“AI in the Loop: Humans in Charge” should be used; ChatGPT widespread use in the near future would be inevitable
Huh [56]	A study to compare ChatGPT performance on a parasitology exam to the performance of Korean medical students	ChatGPT performance will improve by deep learning	ChatGPT performance was lower compared to medical students; plausible explanations for incorrect answers (hallucination)	ChatGPT performance will continue to improve, and health care educators/students are advised to incorporate this tool into the educational process
Khan et al. [57]	A communication on ChatGPT use in medical education and clinical management	ChatGPT can help in automated scoring; assistance in teaching; improved personalized learning; assistance in research; generation of clinical vignettes; rapid access to information; translation; documentation in clinical practice; support in clinical decisions; personalized medicine	Lack of human-like understanding; the limited knowledge up to 2021	ChatGPT is helpful in medical education, research, and in clinical practice; however, the human capabilities are still needed
Gilson et al. [58]	An article on the performance of ChatGPT on USMLE ^3^	Ability to understand context and to complete a coherent and relevant conversation in the medical field; can be used as an adjunct in group learning	The limited knowledge up to 2021	ChatGPT passes the USMLE with performance at a 3rd year medical student level; can help to facilitate learning as a virtual medical tutor
Kung et al. [59]	An article showing the ChatGPT raised accuracy which enabled passing the USMLE	Accuracy with high concordance and insight; it can facilitate patient communication; improved personalized medicine	-	ChatGPT has a promising potential in medical education; future studies are recommended to consider non-biased approach with quantitative natural language processing and text mining tools such as word network analysis
Marchandot et al. [60]	A commentary on ChatGPT use in academic writing	ChatGPT can assist in literature review saving time; the ability to summarize papers; the ability to improve language	Risk of inaccurate content; risk of bias; ChatGPT may lead to decreased critical thinking and creativity in science; ethical concerns; risk of plagiarism	ChatGPT can be listed as an author based on its significant contribution

^1^ WAME: World Association of Medical Editors; ^2^ AI: artificial intelligence; ^3^ USMLE: United States Medical Licensing Examination; ^4^ ICMJE: International Committee of Medical Journal Editors; ^5^ LLM: large-scale language model; ^6^ COPE AI in decision making: Committee on Publication Ethics, Artificial Intelligence (AI) in decision making, available from: https://publicationethics.org/node/50766, accessed on 18 February 2023. * ChatGPT generative pre-trained transformer was listed as an author.

**Table 3:** A summary of the main conclusions of the included records representing preprints.

Author(s) [Record]	Design, Aims	Applications, Benefits	Risks, Concerns, Limitations	Suggested Action, Conclusions
Wang et al. [61]	An arXiv preprint ^1^; investigating ChatGPT effectiveness to generate Boolean queries for systematic literature reviews	Higher precision compared to the current automatic query formulation methods	Non-suitability for high-recall retrieval; many incorrect MeSH ^11^ terms; variability in query effectiveness across multiple requests; a black-box application	A promising tool for research
Borji [20]	An arXiv preprint; to highlight the limitations of ChatGPT	Extremely helpful in scientific writing	Problems in spatial, temporal, physical, psychological and logical reasoning; limited capability to calculate mathematical expressions; factual errors; risk of bias and discrimination; difficulty in using idioms; lack of real emotions and thoughts; no perspective for the subject; over-detailed; lacks human-like divergences; lack of transparency and reliability; security concerns with vulnerability to data poisoning; violation of data privacy; plagiarism; impact on the environment and climate; ethical and social consequences	Implementation of responsible use and precautions; proper monitoring; transparent communication; regular inspection for biases, misinformation, among other harmful purposes (e.g., identity theft)
Cotton et al. [62]	An EdArXiv ^2^ preprint on the academic integrity in ChatGPT era	-	Risk of plagiarism; academic dishonesty	Careful thinking of educational assessment tools
Gao et al. [63]	A bioRxiv ^3^ preprint comparing the scientific abstracts generated by ChatGPT to original abstracts	A tool to decrease the burden of writing and formatting; it can help to overcome language barriers	Misuse to falsify research; risk of bias	The use of ChatGPT in scientific writing or assistance should be clearly disclosed and documented
Polonsky and Rotman [64]	An SSRN ^4^ preprint on listing ChatGPT as an author	ChatGPT can help to accelerate the research process; it can help to increase accuracy and precision	Intellectual property issues if financial gains are expected	AI ^12^ can be listed as an author in some instances
Aczel and Wagenmakers [65]	A PsyArXiv ^5^ preprint as a guide of transparent ChatGPT use in scientific writing	-	Issues of originality, transparency issues	There is a need to provide sufficient information on ChatGPT use, with accreditation and verification of its use
De Angelis et al. [66]	An SSRN preprint discussing the concerns of an AI-driven infodemic	ChatGPT can support and expedite academic research	Generation of misinformation and the risk of subsequent infodemics; falsified or fake research; ethical concerns	Carefully weigh ChatGPT possible benefits with its possible risks; there is a need to establish ethical guidelines for ChatGPT use; a science-driven debate is needed to address ChatGPT utility
Benoit [67]	A medRxiv ^6^ preprint on the generation, revision, and evaluation of clinical vignettes as a tool in health education using ChatGPT	Consistency, rapidity and flexibility of text and style; ability to generate plagiarism-free text	Clinical vignettes’ ownership issues; inaccurate or non-existent references	ChatGPT can allow for improved medical education and better patient communication
Sharma and Thakur [68]	A ChemRxiv ^7^ preprint on ChatGPT possible use in drug discovery	ChatGPT can help to identify and validate new drug targets; to design new drugs; to optimize drug properties; to assess toxicity; and to generate drug-related reports	Risk of bias or inaccuracies; inability to understand the complexity of biologic systems; transparency issues; lack of experimental validation; limited interpretability; limited handling of uncertainty; ethical issues	ChatGPT can be a powerful and promising tool in drug discovery; however, its accompanying ethical issues should be addressed
Rao et al. [69]	A medRxiv preprint on the usefulness of ChatGPT in radiologic decision making	ChatGPT showed moderate accuracy to determine appropriate imaging steps in breast cancer screening and evaluation of breast pain	Lack of references; alignment with user intent; inaccurate information; over-detailed; recommending imaging in futile situations; providing rationale for incorrect imaging decisions; the black box nature with lack of transparency	Using ChatGPT for radiologic decision making is feasible, potentially improving the clinical workflow and responsible use of radiology services
Antaki et al. [70]	A medRxiv preprint assessing ChatGPT’s ability to answer a diverse MCQ ^8^ exam in ophthalmology	ChatGPT currently performs at the level of an average first-year ophthalmology resident	Inability to process images; risk of bias; dependence on training dataset quality	There is a potential of ChatGPT use in ophthalmology; however, its applications should be carefully addressed
Aydın and Karaarslan [71]	An SSRN preprint on the use of ChatGPT to conduct a literature review on digital twin in health care	Low risk of plagiarism; accelerated literature review; more free time for researchers	Lack of originality	Expression of knowledge can be accelerated using ChatGPT; further work will use ChatGPT in citation analysis to assess the attitude towards the findings
Sanmarchi et al. [72]	A medRxiv preprint evaluating ChatGPT value in an epidemiologic study following the STROBE ^9^ recommendations	ChatGPT can provide appropriate responses if proper constructs are developed; more free time for researchers to focus on experimental phase	Risk of bias in the training data; risk of devaluation of human expertise; risk of scientific fraud; legal issues; reproducibility issues	Despite ChatGPT possible value, the research premise and originality will remain the function of human brain
Duong and Solomon [73]	A medRxiv preprint evaluating ChatGPT versus human responses to questions on genetics	Generation of rapid and accurate responses; easily accessible information for the patients with genetic disease and their families; it can help can health professionals in the diagnosis and treatment of genetic diseases; it could make genetic information widely available and help non-experts to understand such information	Plausible explanations for incorrect answers (hallucination); reproducibility issues	The value of ChatGPT will increase in research and clinical settings
Yeo et al. [74]	A medRxiv preprint evaluating ChatGPT responses to questions on cirrhosis and hepatocellular carcinoma	Improved health literacy with better patient outcome; free availability; increased efficiency among health providers; emulation of empathetic responses	Non-comprehensive responses; the limited knowledge up to 2021; responses can be limited and not tailored to specific country or region; legal issues	ChatGPT may serve as a useful aid for patients besides the standard of care; future studies on ChatGPT utility are recommended
Bašić et al. [75]	An arXiv preprint on the performance of ChatGPT in essay writing compared to masters forensic students in Croatia	-	Risk of plagiarism; lack of originality; ChatGPT use did not accelerate essay writing	The concerns in the academia towards ChatGPT are not totally justified; ChatGPT text detectors can fail
Hisan and Amri [76]	An RG ^10^ preprint on ChatGPT use medical education	Generation of educational content; useful to learn languages	Ethical concerns; scientific fraud (papermills); inaccurate responses; declining quality of educations with the issues of cheating	Appropriate medical exam design is needed, especially for practical skills
Jeblick et al. [77]	An arXiv preprint on ChatGPT utility to simplify and summarize radiology reports	Generation of medical information relevant for the patients; moving towards patient-centered care; cost efficiency	Bias and fairness issues; misinterpretation of medical terms; imprecise responses; odd language; hallucination (plausible yet inaccurate response); unspecific location of injury/disease	Demonstration of the ability of ChatGPT simplified radiology reports; however, the limitations should be considered. Improvements of patient-centered care in radiology could be achieved via ChatGPT use
Nisar and Aslam [78]	An SSRN preprint on the assessment of ChatGPT usefulness to study pharmacology	Good accuracy	Content was not sufficient for research purposes	ChatGPT can be a helpful self-learning tool
Lin [79]	A PsyArXiv preprint to describe ChatGPT’s utility in academic education	Versatility	Hallucination (inaccurate information that sounds scientifically plausible); fraudulent research; risk of plagiarism; copyright issues	ChatGPT has a transformative long-term potential; embrace ChatGPT and use it to augment human capabilities; however, adequate guidelines and codes of conduct are urgently needed

^1^ arXiv: A free distribution service and an open-access archive for scholarly articles in the fields of physics, mathematics, computer science, quantitative biology, quantitative finance, statistics, electrical engineering and systems science, and economics, materials on arXiv are not peer-reviewed by arXiv, available from: https://arxiv.org/, accessed on 18 February 2023; ^2^ EdArXiv: A preprint server for the education research community, available from: https://edarxiv.org/, accessed on 19 February 2023; ^3^ bioRxiv: A free online archive and distribution service for unpublished preprints in the life sciences, available from: https://www.biorxiv.org/, accessed on 19 February 2023; ^4^ SSRN: Social Science Research Network repository for preprints, available from: https://www.ssrn.com/index.cfm/en/, accessed on 19 February 2023; ^5^ PsyArXiv: Psychology archive for preprints, available from: https://psyarxiv.com/, accessed on 18 February 2023; ^6^ medRxiv: Free online archive and distribution server for complete but unpublished manuscripts (preprints) in the medical, clinical, and related health sciences, available from: https://www.medrxiv.org/, accessed on 18 February 2023; ^7^ ChemRxiv is a free submission, distribution, and archive service for unpublished preprints in chemistry and related areas, available from: https://chemrxiv.org/engage/chemrxiv/public-dashboard, accessed on 18 February 2023; ^8^ MCQ: Multiple choice exam; ^9^ STROBE: Strengthening the reporting of observational studies in epidemiology; ^10^ RG: ResearchGate: A commercial social networking site for scientists and researchers, available from: https://www.researchgate.net/about, accessed on 19 February 2023; ^11^ MeSH: Medical Subject Headings; ^12^ AI: Artificial intelligence.

## Data Availability

Data supporting this systematic review are available in the original publications, reports, and preprints that were cited in the reference section. In addition, the analyzed data that were used during the current systematic review are available from the author on reasonable request.
